# Impact of mitochondrial dysfunction on the antitumor effects of immune cells

**DOI:** 10.3389/fimmu.2024.1428596

**Published:** 2024-10-11

**Authors:** Quan Wang, Xiangzhi Yin, Xiaotong Huang, Lu Zhang, Haijun Lu

**Affiliations:** ^1^ Department of Radiation Oncology, The Affiliated Hospital of Qingdao University, Qingdao, China; ^2^ Department of Orthopaedics, Affiliated Hospital of Qingdao University, Qingdao, China; ^3^ Department of Radiation Oncology, The Affiliated Hospital of Shandong University of Traditional Chinese Medicine, Jinan, China

**Keywords:** mitochondrial dysfunction, immunotherapy, immune regulation, tumor microenvironment, immune cell

## Abstract

Mitochondrial dysfunction, a hallmark of immune cell failure, affects the antitumor effects of immune cells through metabolic reprogramming, fission, fusion, biogenesis, and immune checkpoint signal transduction of mitochondria. According to researchers, restoring damaged mitochondrial function can enhance the efficacy of immune cells. Nevertheless, the mechanism of mitochondrial dysfunction in immune cells in patients with cancer is unclear. In this review, we recapitulate the impact of mitochondrial dysfunction on the antitumor effects of T cells, natural killer cells, dendritic cells, and tumor-associated macrophage and propose that targeting mitochondria can provide new strategies for antitumor therapy.

## 1 Introduction

Cancer is the leading cause of mortality in both industrialized and developing countries, generating severe concerns about global public health (1). Following the approval of the first immune checkpoint inhibitor, ipilimumab, for clinical use in 2011, immunotherapy for cancer has gradually gained momentum, markedly improving the survival rate of patients with cancer (2). However, despite remarkable advancements in immunotherapy, approximately 50% of patients do not benefit from it. There remain several limitations to immunotherapy, such as biomarker identification, combination of immunotherapy with other treatment methods, immune escape, and optimization of treatment endpoint evaluation. This highlights the broad link between cancer and the immune system, underscoring the inherent complex variability of the immune system (3). Exploring antitumor immunity is crucial to achieving long-term survival through immunotherapy in patients with cancer.

Mitochondria, which, according to the endosymbiotic theory, originated from α-proteobacteria, are widely recognized as the “energy factory” of eukaryotic cells (4). Saha et al. have demonstrated that the anticancer activities of T cells were increased by the combination of a dual farnesyl transferase and geranylgeranyl transferase inhibitor (L-778,123) targeting nanotube production with anti-PD-1 antibody (5). Further, they revealed that tumor cells acquire mitochondria from immune cells through nanotubes, prolonging their own viability while inactivating immune cells, thus evading immune surveillance. In the past century, the impact of mitochondrial dysfunction on tumor onset, metastasis, and progression has been clarified, and mitochondria-targeting has been demonstrated as an effective strategy for antitumor therapy (6). As more and more patients benefit from immunotherapy, the significance of the functional status of mitochondria in immune cells for immunotherapy efficacy in patients with cancer has garnered growing attention among researchers. The functions of immune cells are impacted by dynamic changes in mitochondria, such as fission, fusion, and metabolism, and enhancing mitochondrial function improves the efficacy of immunotherapy (7–9). This review focuses on altered mitochondrial function in immune cells of patients with cancer and explores treatment strategies targeting mitochondrial dysfunction to strengthen the antitumor activities of immune cells.

## 2 Mitochondria in immune cells

Mitochondria are organelles composed of two phospholipid membranes, the outer mitochondrial membrane (OMM) and inner mitochondrial membrane (IMM). The main function of the OMM, which has a high permeability, is to exchange substances between mitochondria and cytoplasm or endoplasmic reticulum. The IMM has low permeability and folds inward to form cristae, the main site of cellular energy conversion. Cristae area determines the rate of mitochondrial oxidative phosphorylation (OXPHOS) (10). Mitochondrial metabolism has three main functions: (і) catabolism, which involves breakdown of nutrients (sugar, protein, and fat) to generate adenosine triphosphate (ATP) for cell survival through the electron respiratory chain coupled with oxidative phosphorylation; (II) anabolism, in which the tricarboxylic acid cycle (TCA) metabolites in mitochondria are used for the synthesis of macromolecules; and (III) production of signaling molecules that control the functional activities of cells, such as reactive oxygen species (ROS) and cytochrome c (11). As a dynamic organelle, mitochondria can undergo morphological adaptations and interconvertibly assume the appearance of long and short tubules. Mitochondria undergo morphological changes such as fission and fusion to adapt to their living environment in cells, which is called mitochondrial dynamics (12). Mitochondrial fusion involves the fusion of the OMM and IMM (13). Briefly, IMM fusion is drived by mitofusin 1 and mitofusin 2, and OMM fusion by optic atrophy 1 protein (OPA1), which provides a structural basis for cellular anabolism (14). Dynamin-related protein 1 (DRP1) is the mediator of mitochondrial fission and is essential for preserving the quantity of mitochondria, encouraging their movement, and separating damaged mitochondria (15). Mitochondria are transported in the cytoplasm by microtubules and actin, depending on the cellular metabolic needs. Mitophagy, the particular deterioration of damaged mitochondria, is drived by the PINK/Parkin pathway, which is essential for maintaining mitochondrial homeostasis and function (16).

Mitochondrial morphology and energy metabolism are serially connected (17). For example, in T cell differentiation, immature T cells (Tn) in a long-term quiescent state rely on the TCA cycle and OXPHOS for oxidative capacitation. When activated by antigens and other substances, Tn rapidly differentiates into effector T cells (Te), and mitochondria respond immediately. The metabolic reprogramming of mitochondria through fission promotes anabolism and a metabolic shift to glycolysis and glutaminolysis (18). This metabolic pattern in immune cells, in which glycolysis is favored over OXPHOS with the existence of sufficient oxygen, is similar to the Warburg effect in tumor cells (19). Although the general consensus is that mitochondrial metabolic reprogramming in immune cells occurs because these cells use the byproducts of aerobic glycolysis for anabolism, some experts believe that immune cells synthesize ATP through aerobic glycolysis at a higher rate than through OXPHOS; however, these views are yet to be fully clarified (20). Meanwhile, some studies have shown that various T cell metabolic activities influence mitochondrial structure (21). More studies are needed to confirm the relationship between mitochondrial morphology and metabolism.

Thus, mitochondria in immune cells involved in anti-tumor responses actively respond to changes of cells and the body, constantly changing their morphology and position to achieve metabolic optimization, while also being influenced by metabolism to maximize their functions.

## 3 T cell

**Figure 1:**
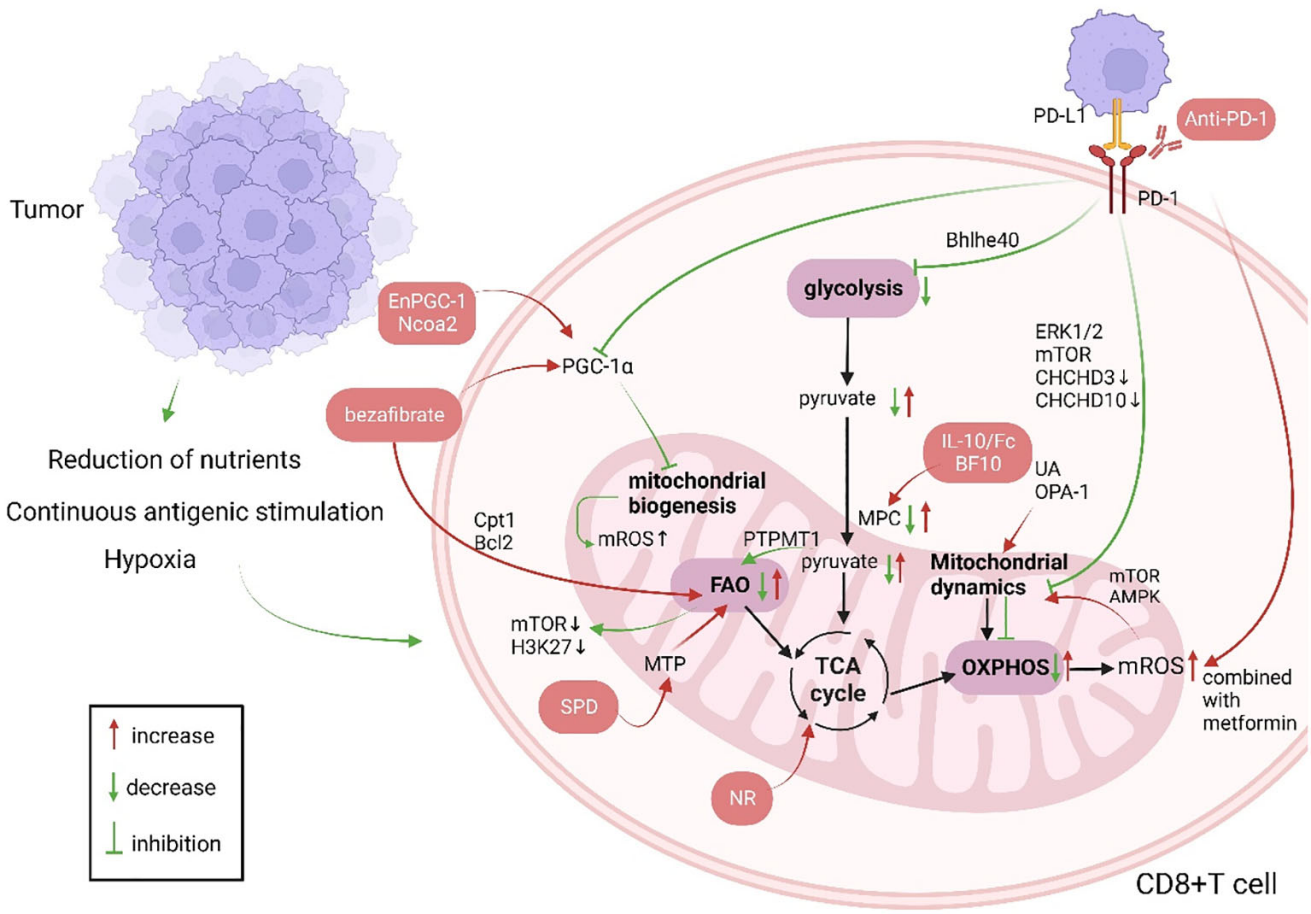
Changes in CD8+ T cell mitochondrial function in patients with cancer and anti-tumor immunotherapy strategies targeting CD8+ T cell mitochondria (green indicates mitochondrial changes in patients with cancer, red indicates cellular changes after immunotherapy, and red boxes indicate treatment strategies). Produced with the aid of BioRender (http://biorender.com) (1). The long-term tumor microenvironment poses a threat to CD8+ T cells, which is manifested as lack of nutrients, hypoxia, and long-term stimulation of tumor antigens. (2) PGC-1α affects mitochondrial biogenesis, and UA and OPA-1 affect mitochondrial dynamics, so that the mitochondrial mass and morphology can not meet the needs of cell metabolism. When CD8+ T cells are activated, their metabolism mainly shifts to glycolysis, and OXPHOS is inhibited. Increased FAO leads to energy imbalance and ROS production. PD-1 not only affects mitochondrial metabolism but also mitochondrial dynamics through Drp1. (3) Immunotherapies targeting mitochondria mainly involve supplementing metabolites to restore metabolic activity and improved mitochondrial dynamics, combining immunosuppressive agents, etc. Ncoa2, nuclear receptor coactivator 2; Bcl2, B-cell lymphoma-2; PGC-1α, peroxisome proliferator activated receptor gamma coactivator-1alpha; ROS, reactive oxygen species; MTP, mitochondrial trifunctional protein; SPD, spermidine; PTPMT1, protein tyrosine phosphatase mitochondrial 1; TCA, tricarboxylic acid cycle; IL-10, interleukin-10; UA, urine acid; OPA-1, optic atrophy 1; OXPHOS, oxidative phosphorylation; FAO, fatty acid oxidation; mTOR, mammalian target of rapamycin; PD-1, programmed cell death 1; PD-L1, programmed death ligand 1; ERK1/2, extracellular regulated protein kinases; CHCHD, coiled-coil-helix-coiled-coil-helix domain; AMPK, Adenosine 5’-monophosphate (AMP)-activated protein kinase; Cpt1, Carnitine palmitoyl transferase 1; Bhlhe40, basic helix-loop-helix family member e40;.

Upon recognition of antigens presented by major histocompatibility complex (MHC) and costimulation, T cells undergo positive and negative selection within the thymus and differentiate into CD4+ T cells and CD8+ T cells. While CD4+ T cells serve as T helper cells, CD8+ T cells are cytotoxic T cells. Most research concentrates on the antitumor activities of CD8+ T cells (22, 23). Reduced responses to immune checkpoint inhibitors and to adoptive T cell therapy in patients are closely related to T cell exhaustion, which is a condition of progressive decline in T cell function due to persistent antigenic stimulation in the tumor microenvironment or viral. T cell exhaustion is distinguished by boost in the expression of T cell inhibitory receptors (including LAG-3, PD-1, and CTLA-4), decreased effector function, metabolic reprogramming, and altered epigenetic landscape (7, 24, 25). Mitochondrial function influences T-cell exhaustion (26) (**Figure 1**).

### 3.1 Mitochondrial biogenesis

Mitochondrial biogenesis increases mitochondrial mass and promotes metabolism to maintain mitochondrial function. Peroxisome Proliferator-Activated Receptor Gamma Coactivator 1-alpha (PGC-1α), a crucial component of the mitochondrial biogenesis signaling cascade, not only promotes central memory T cell (Tcm) formation but also improves the structural and metabolic adaptability of CD8+ T cell mitochondria in tumor microenvironment (TME). PGC-1α was used as a “transfer station”. The processes of cellular differentiation, metabolism, structure, and Ca2+ transport are serially connected to persist the basic function of CD8+ T cells in the TME (26, 27). Scharping reported CD8+ T cell depletion in an *in vitro* tumor model mimicking hypoxia and continuous antigen stimulation. Further, they found that continuous antigen stimulation enhanced Blimp-1-mediated inhibition of PGC-1α in mitochondrial biogenesis and adaptation. This leads to mitochondrial failure, which generates ROS that cannot be eliminated, inhibits phosphatase, and enhances Nuclear Factor of Activated T cell (NFAT) activity (26, 28). In non-small cell lung cancer, MHC-II expressing antigen-presenting cancer-associated fibroblasts evade immune cell-mediated apoptosis by antigen-stimulated production of C1q, which interacts with the complement C1q binding protein (C1QBP) on CD4+ T cells, impairing their antitumor effects and contributing to tumor progression (29). C1QBP is a mitochondrial protein, the deletion of which increases mitochondrial fission through Adenosine 5’-monophosphate-activated protein kinase (AMPK)/PGC-1α signaling and inhibits mitochondrial biogenesis, further impairing mitochondrial plasticity and metabolism; this affects the number, depletion, and memory phenotype of tumor-infiltrating lymphocytes (TILs) (30). However, whether other signaling pathways of CD8+ TILs in lung cancer and other tumors are affected by AMPK/PGC-1α signaling remains unclear. Moreover, determining whether PGC-1α is a single factor is difficult.

### 3.2 Mitochondrial dynamics

In the TME, changes in mitochondrial dynamics not only affect the metabolism of CD8+ T cells, but also cell migration (31). Mitochondrial morphology adapts to changes in cellular functional status, and changing the morphology of cristae makes fused mitochondria more conducive to OXPHOS, as in Tn cells with fragmented and round mitochondria (32–34). Regulating mitochondrial fission and fusion in CD8+ T cells is important for adaptation to TME. BCL11B knockout in T cells can upregulate OPA1 expression and mediate mitochondrial fusion. Elongated mitochondria promote OXPHOS to increase energy production in T cells (35). High levels of Drp1, which mediates mitochondrial cleavage, can be used in combination with PD-1 inhibitors to improve anti-tumor effects. This may be the result of the PD-1-ERK/Drp1 pathway as well as cytokine secretion (36). Knockdown of Drp1 reveals fused mitochondria and suppresses T cell migration (37). Interestingly, Simula et al. suggest that PD-1-mediated downregulation of Drp1 does not regulate metabolism. Due to the heterogeneity and diversity of tumors, the mechanisms by which mitochondrial morphology changes in tumor-infiltrating CD8+ T cells remain unclear.

### 3.3 Mitochondrial metabolism

In the TME, upon continuous activation by T cell receptors, CD8+ T cells undergo a metabolic shift to glycolysis and glutaminolysis to maintain their antitumor functions. However, tumor cells compete with immune cells for nutrients, such as sugar and oxygen, inhibiting metabolism in immune cells (28, 38–40). Glycolysis provides pyruvate to the TCA cycle, the main pathway of mitochondrial energy production. However, tumor cells influence pyruvate utilization in many ways (41). Mitochondrial OXPHOS rate affects the antitumor efficacy of CD8+ T cells. In glutamine- and fatty acids-deficient TME, CD8+ T cells with suppressed mitochondrial pyruvate carrier rely on lactate oxidation, which leads to mammalian target of rapamycin (mTOR) inactivation and H3K27 methylation, impairing their antitumor function (42, 43). Chen et al. demonstrated that the loss of protein tyrosine phosphatase mitochondrial 1 in CD8+ T cells inhibited the oxidative utilization of pyruvate by mitochondria, resulting in elevated FAO rate; this change in metabolic substrate and metabolic inflexibility accelerated T cell failure (44). Lactate accumulation and the lack of glucose and fatty acids in the TME cause an imbalance in the metabolic microenvironment. In addition, chronic antigen stimulation impairs OXPHOS and reduces ATP production (45).

### 3.4 Immune checkpoint signaling

At present, an increasing amount of research have shown that immune checkpoint signal transduction can affect mitochondrial function, mainly mitochondrial metabolism and dynamics (26). T cell failure is characterized by persistent PD-1 expression. In the early stage of failure, PD-1 signaling inhibits PGC-1α, resulting in metabolic disorders, excessive mtROS production, and increased number of depolarized mitochondria (46, 47). PD-1 signaling also suppresses the manifestation of the transcription factor basic helix-loop-helix family member e40 (Bhlhe40), which primarily affects mitochondrial metabolism and fitness (48). PD-1 signal transduction inhibits the motility and proliferation of T cells by regulating extracellular signal-regulated kinase 1/2 (ERK1/2) and mTOR proteins to phosphorylate Drp1 on Ser616, preventing its activation (37). Moreover, PD-1 signaling leads to a decrease in mitochondrial cristae length and quantity, affecting their glycolysis and OXPHOS rates by reducing Coiled-Coil-Helix-Coiled-Coil-Helix Domain Containing 3 (CHCHD3) and CHCHD10 expression (49).

### 3.5 The endoplasmic reticulum-mitochondria interface

There is a growing consensus that the association between endoplasmic reticulum (ER) and mitochondria is not only related to the T cell’s activation and differentiation, but also to their exhaustion (50–52). First, the mitochondria-ER contact site (MERC) in TILs is mediated by MFN2-sarcoplasmic/endoplasmic reticulum Ca2+ ATPase 2 (SERCA2) interaction to regulate mitochondrial Ca2+ homeostasis and maintain T cell metabolism and adaptability (53). High cholesterol metabolism increases the manifestation of mitophagy-related proteins (PINK, BNIP3, and Parkin) in CD8+ T cells in patients with colorectal cancer through the ERS-ERMC-mitophagy axis, which inhibits mitochondrial metabolism (54). In the TME, ER stress cannot be avoided by regulating MERC to promote mitochondrial fission, fusion, autophagy, or Ca2+ influx and maintain constant energy in TILs (52). Protein kinase RNA-like ER kinase-mediated induction of mtROS is a marker of mitochondrial failure in CD8+TIL. ER stress in ovarian cancer-activated T cells inhibits glutamine influx via unfolded protein response. Thus, the effect of tumor cells on tandem organelles in TILs is mediated not only through the MERC interface but also through other molecules, providing new targets for immunotherapy (52, 55, 56).

## 4 Natural killer cells

Natural killer (NK) cells are crucial components of the innate immune system (57); these cells can kill tumor cells through MHC-I and antibody-dependent direct cytotoxic effects. They can also release cytokines, such as IL-8 and macrophage inflammatory protein -1α, to play an immunomodulatory role in promoting antitumor functions of T and B cells (58, 59). Although the current classical phenotype of NK cells is not sufficient to further classify them according to different functions, mitochondrial metabolic activity can be used as a basis for distinguishing them: cytotoxic NK cells exhibit enhanced ability to metabolize glucose through OXPHOS and glycolysis; regulatory NK cells continue to function under hypoxia and limiting glycolysis; memory NK cells maintain improved fitness of the mitochondria by eliminating dysfunctional mitochondria to increase membrane potential and respiratory capacity of mitochondria and reduce ROS levels (60). NK cells exhibit a long memory phenotype, and there are many antitumor mechanisms that need to be explored in adaptive immunity (61). O’Sullivan et al. showed that NK cells acquired immunological memory through mitophagy mediated by the mitochondria-associated proteins BNIP3 and BNIP3L (62).

**Figure 2:**
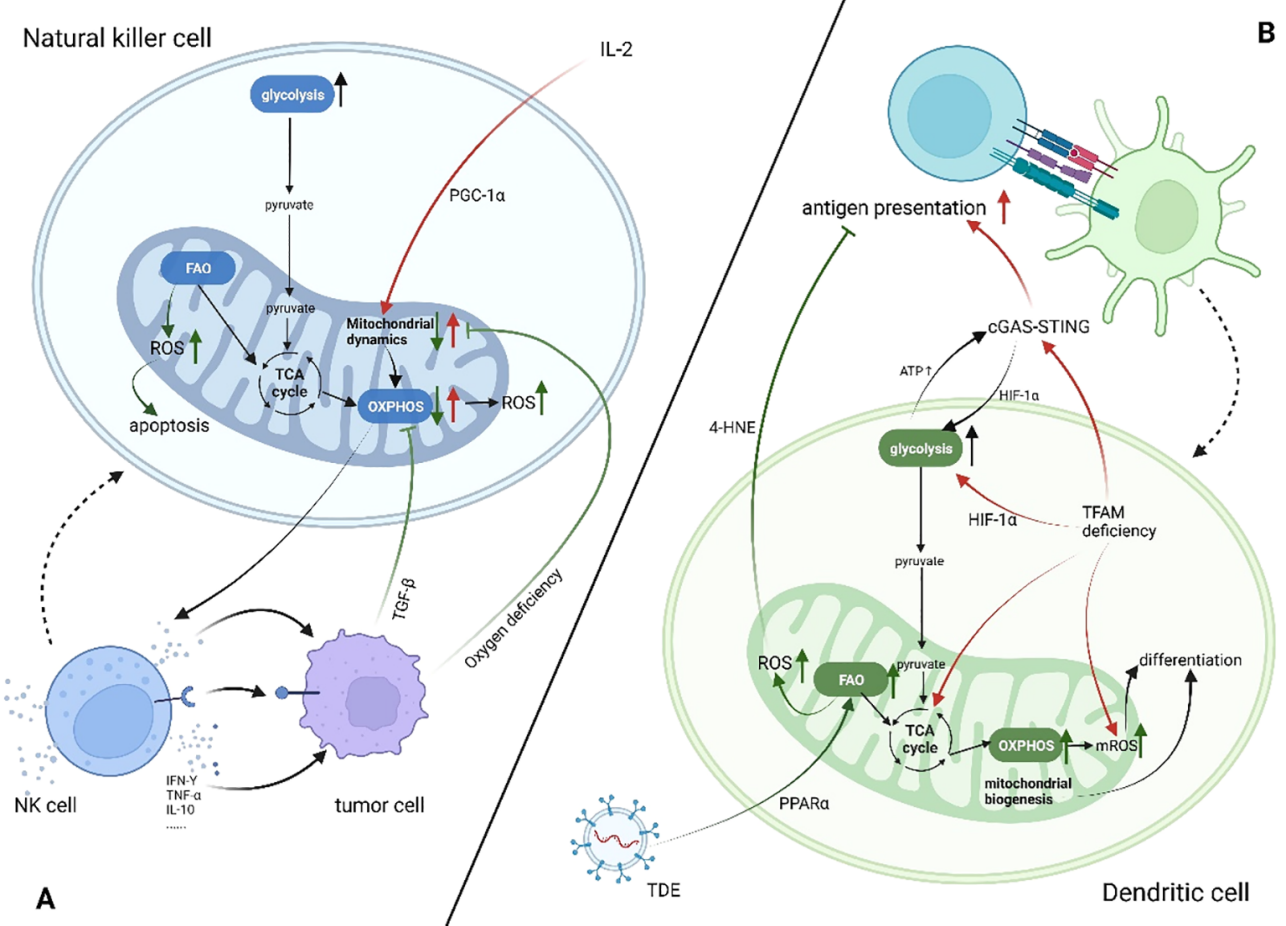
Mitochondrial changes in dendritic cells and natural killer cells in patients with cancer (increase↑, decrease↓). Created using BioRender software (http://biorender.com). **(A)** Tumor cells secrete cytokines such as TGF-β or compete for oxygen, which leads to the inhibition of OXPHOS in NK cells, mitochondrial cleavage, and insufficient energy supply. At the same time, ROS production increases, promoting cell apoptosis and inhibiting the antitumor effect of NK cells. **(B)** In the TME, both FAO and glycolysis in DC cells are enhanced but have different effects on DC antigen presentation. IL-2, interleukin-2; TGF-β, transforming growth factor β; TNF-α, tumor necrosis factor α; IFN-γ, interferon γ; cGAS-STING, the cyclic GMP-AMP synthase -sstimulator of interferon genes pathway; HIF-1α, hypoxia-inducible factor alpha; TFAM, transcription factor a, mitochondrial; TDE, tumor-derived exosome; 4-HNE, 4-hydroxynonenal; ATP, adenosine 5’-triphosphate.

Mitochondria play a significant part in the antitumor activity of NK cells, mainly by affecting NK cells’ metabolism. Specifically, mitochondria are the energy supply centers of cells, and the cytotoxic effect of NK cells is activated by the cytokine IL-2, which provides energy for cellular activity (63). Slattery et al. found that when NK cells from patients with neuroblastoma kill tumor cells by antibody-dependent cell-mediated cytotoxicity, increased glycolysis is mediated by mitochondrial dysfunction (64). Transforming growth factor-β (TGF-β) produced by tumor cells and tumor-associated cells directly causes mitochondrial dysfunction by inhibiting mitochondrial respiration and other mechanisms, which leads to NK cells’ metabolic malfunction in patients with breast cancer that has spread (65). Zheng et al. found that hypoxic TME causes continuous activation of mTOR-Drp1 targets in NK cells, leading to an increased rate of fission and fragmentation of the mitochondria; this affects mitochondrial metabolism and antitumor functions of NK cells. Furthermore, they confirmed the connection between mitochondrial dynamics and metabolic reprogramming (66). Apart from impacting metabolism, the products of mitochondria in biological activities have different implications for NK cell antitumor activity. Mitochondria are the main production site of ROS, which is required for NK cell-mediated tumor cell lysis. However, lactic acid-induced ROS also promotes apoptosis in NK cells, and mitochondrial dysfunction with immunosenescence is associated with excessive ROS production (67, 68). Abarca-Rojano et al. proved that the development of NK cell immune synapses is assisted by mitochondria, and that mitochondrial recombination into these synapses based upon activating receptors, such as Natural killer group 2, member D (NKG2D) (69). NK cells develop immune memory through three stages. However, how mitochondria mediate each metabolite in this process is still unclear. Further studies are needed to determine whether metabolic changes can be used to target mitochondria in NK cells, promoting their long-term memory phenotype instead of apoptosis to develop novel antitumor therapy. (**Figure 2**)

## 5 Dendritic cell

Dendritic cells (DCs) are a group of myeloid-derived antigen-presenting cells (APCs) with specialized functions (70). These cells have the ability to combine environmental data and transmit it to other leukocytes, generating both innate and adaptive immunity (71). Mitochondrial dysfunction affects differentiation, metabolism, T cell activation, and antigen presentation of DCs (**Figure 2**). DC differentiation is characterized by upregulation of mitochondrial respiratory complexes and increased mitochondrial DNA copy number, indicating active mitochondrial biogenesis (72). ROS plays a crucial role in the differentiation of DC. Del Prete et al. demonstrate that the treatment of NK cells with rotenone or catalase results in a decrease in ROS production, which leads to the obstruction of DC differentiation (73). ROS content also controls the differentiation of DC subpopulations, and ROS inhibition increases the proportion of cDC1 (74). Mitochondria plays a vital role in antigen processing and presentation by APCs (75). GMP-AMP synthase (cGAS)-stimulator of interferon genes (STING) pathway is a vital innate immune pathway which links innate and adaptive immunity. Recently, it was found that the antitumor effect of this pathway is closely related to mitochondrial function (76). Lu et al. have demonstrated that mitochondrial transcription factor A (TFAM) deficiency in DCs leads to mitochondrial DNA escape, which triggers the cGAS-STING pathway to promote DC migration, maturation, antigen presentation, and secretion of inflammatory factors and restore the anti-tumor effect of DC (77). Hu et al. have reported that DCs in TME exhibited enhanced aerobic glycolysis and ATP production, which promoted STING phosphorylation and STING-dependent antitumor activation of DC. Simultaneously, the intrinsic STING-mediated activation of DC promoted hypoxia-inducible factor-1 alpha (HIF-1α)-mediated glycolysis to establish a positive feedback pathway against tumor cells (78). It is crucial for DC tolerance to be maintained that glycolysis occurs. In the TME, tumor-derived exosomes carrying fatty acids or tumor cells use the paracrine Wnt5a/b-catenin signaling pathway to activate peroxisome proliferator-activated receptors in DC cells, leading to enhanced FAO. Consequently, mitochondrial metabolism is switched to OXPHOS, which leads to DC immune dysfunction (79–81). This may be due to increased ROS production by mitochondria through FAO rather than glycolysis, which subsequently leads to the production of peroxidation byproducts, consisting of 4-hydroxynonenal (4-HNE), preventing antigen cross-presentation (82). However, ROS production by mitochondria is also essential to the presentation and processing of antigens by APC. Antigen cross-presentation to CD8+ T cells by Plasmacytoid DCs is dependent on mitochondrial ROS production and involves pH alkalinization and antigen protection (83). Meanwhile, DC-mediated activation of Tn cells requires mitochondria to maintain their integrity (84). However, most studies on DC tend to focus on the functional changes of mitochondria when T cells are activated, and few have focused on mitochondrial dysfunction in DC cells in the TME.

## 6 Tumor-associated macrophages

Tumor-associated macrophages (TAMs) are the predominant kind of macrophages seen in TME (85). TAM can be divided into M1 pro-inflammatory anti-tumor type and M2 anti-inflammatory pro-tumor type (86). This, however, is not a straightforward choice. There are also many intermediate types of Tams that express markers of both M1 and M2 (87). Nonetheless, the types of TAM mentioned in TME is primarily of M2 macrophage, and excessive TAM infiltration is linked to a poor patient prognosis (88). ROS production occurs mostly in mitochondria and is intimately associated with TAM polarization (89). ROS can also be secreted by cancer cells.

Studies show that M1 macrophages are pro-inflammatory and secrete a large number of ROS and inflammatory factors. ROS is a necessary condition for the phenotype polarization of TAM towards M2, which is conducive to the formation of immunosuppressive TME (90–92). However, the ROS content in M2 macrophages was lower. This is why M2 macrophages have lower concentrations of pro-oxidants like NOX and higher concentrations of antioxidant enzymes like catalase and Gpx1 (93). It has now been demonstrated that ROS contributes to M1 polarization as well. Two DNA binding agents, trabectedin (TRB) and lurbinectedin (LUR), activated the PPP pathway upon TAM, promoted ROS production, and exhibited an M1-polarized phenotype (94). This implies that TAM polarization may be impacted by REDOX targeting of TAM. Liang et al. design a new nanomaterial SV@BMs to generate a large number of ROS in TAM. ROS inhibits cancer cell growth in TME and promotes the polarization of TAM to M1 through the NF-κB pathway together with manganese ions (95). Shi et al. design a novel nanoparticle using photodynamic release of ROS, which promotes the polarization of the M2 type into the M1 type through the NF-κB pathway. In order to prevent the toxic effects of ROS, NH4HCO3 was added to the nanomaterials to control the production of ROS (96). Cu2-xSe nanoparticles have the ability to generate ROS in TAM, ubiquitinate TRAF6, stimulate IRF5 to increase IL-5 production, and polarize M2 to M1 (97). The synergistic effect of ROS with ZnPP PM/PIC causes M2 TAM to redevelop the M1 phenotype. It’s probable that ZnPP PM/PIC works on TAM to suppress STAT3 expression through ROS (98). There are more factors influencing TAM polarization than ROS. The different polarization results of ROS to TAM may be due to the combined effects of the amount of ROS, the signal pathway activated by ROS and the activation of the signal pathway related to ROS. More research is necessary to determine whether ROS has an antitumor or antitumor function in tumor therapy.

## 7 Antitumor therapy targeting T cell mitochondria

### 7.1 Targeting the PGC-1α signaling pathway

Owing to its function, PGC-1α has attracted attention as a target for antitumor therapy, including its potential to enhance the efficacy of existing immunotherapies or adoptive cell therapy (ACT) (99). PGC-1α/peroxisome proliferator-activated receptor complex agonists can activate mitochondria, increase OXPHOS and glycolysis, increase fatty acid oxidation (FAO) via carnitine palmitoyltransferase 1 and B-cell lymphoma-2 (Bcl2) upregulation, and improve CD8+ T cell effector function, enhancing its antitumor effect when used in combination with PD-1 inhibitors. Malinee et al. developed EnPGC-1, an epigenetic activator of PGC-1α/β based on DNA (100, 101). The nuclear receptor coactivator 2 (NCOA2)-based therapy developed by Zhong et al. is also based on PGC-1α. Mice lacking Ncoa2 in T cells are unable to stimulate PGC-1α expression, and therefore have lower mitochondrial mass, reduced interferon-γ production, and impaired OXPHOS, with faster tumor growth and progression (102). However, PCG-1α is neither affected by a single pathway, nor does it affect a single downstream target. Mitochondrial biogenesis-based treatment combined with immunotherapy has shown advantages, and there is an urgent need to clarify the underlying mechanism.

### 7.2 Targeting mitochondrial metabolism

Mitochondrial function can be restored to produce sufficient energy for resisting attacks by tumor cells through metabolic reprogramming or substrate supplementation, depending on the type of mitochondrial metabolism imbalance, i.e., substrate reduction or impaired metabolite production during glycolysis or OXPHOS. Siska et al. found that glycolysis and mitochondrial function in TILs are impaired in clear cell renal cell carcinoma, and supplementation with pyruvate or mitochondrial ROS scavengers can partially restore CD8+ T cell function (40). Spermidine can improve low response rate to PD-1 therapy in elderly patients with cancer by directly combining with mitochondrial trifunctional protein to promote FAO (103). 5-ALA/SFC leads to PGC-1α upregulation through Nuclear Respiratory Factor 1 (Nrf-1), Heme Oxygenase-2 (HO-2), Sirtuin 1 (Sirt-1), and PGC-1α pathways, which enhances mitochondrial function *in vitro* and increases oxygen consumption rate, elevating the production of complex V and ATP, which are required for the induction of effector ZCD8+ T cells (104). Guo et al. developed an interleukin (IL)-10-Fc fusion protein to modulate metabolism and promote OXPHOS recovery in terminally depleted CD8+ TILs through a new material technology. IL-10 was fused with a colony-stimulating factor-1 receptor blocking antibody to generate a bifunctional protein for metabolic reprogramming (105, 106). However, CD8+ T cells have different energy requirements with different stimuli in the TME. Therefore, the treatment should reprogram mitochondrial metabolism in order to fulfill the demands of cell differentiation and cell metabolism according to the patient’s autoimmune status, which is still rarely studied.

### 7.3 Targeting PD-1 inhibitors

At present, treatment with PD-1 inhibitors is accompanied by supplementation with nicotinamide nucleoside to restore mitochondrial metabolism (47). Combination therapy with PD-1 inhibitors and metformin can stimulate mtROS production and promote CD8+ T cell proliferation, likely due to the promotion of downstream mTOR and AMPK by ROS (107, 108). It also has synergistic effects when combined with costimulatory receptors, costimulatory molecules, and cytokines, such as 4–1BB agonists, IL15, IL2, and IL10 (109–111).

## 8 Conclusion

Mitochondria function as a transfer station in biological processes of immune cells, playing essential parts in both energy supply and cell migration. Immune cell failure in patients with cancer is marked by mitochondrial dysfunction. Therefore, mitochondria represents a therapeutic target to restore immune function. In this review, we explored the effects of mitochondrial dysfunction on the antitumor functions of T cells, NK cells, DC cells and TAMs. We propose that tumor cells impact mitochondria in immune cells since their stimulation by tumor antigens. Moreover, mitochondria play crucial roles in the antitumor functions of immune cells by regulating metabolism and kinetics. However, if the antitumor response is inadequate, tumor cells take advantage and lead to tumor progression and shortened overall survival. We summarize ways to target T cell mitochondria to improve antitumor therapy. Mitochondrial biogenesis can be activated by targeting the PGC-1α signaling pathway. Supplement the products required for mitochondrial metabolism and promote mitochondrial metabolic reprogramming. Activation of mitochondrial function in combination with PD-1 inhibitors. We summarize methods that can be combined with existing tumor treatment methods, such as chimeric antigen receptor T-cell therapy, immunotherapy, radiotherapy, chemotherapy, and surgery. However, most existing studies are in the preclinical stage, and few have progressed to clinical trials. To fully evaluate the effectiveness and safety of such treatment plans in clinical settings, more research is required. Due to the heterogeneity of tumors and the diversity of individuals, the responses of immune cell mitochondria to changes in the TME are holistic and cannot be analyzed separately, such as changes in mass, volume, or metabolic pathway. Future studies should explore how to avoid or reverse mitochondrial dysfunction in immune cells and target mitochondrial function to ultimately improve clinical outcomes in patients.
